# Predicting Total Back Squat Repetitions from Repetition Velocity and Velocity Loss

**DOI:** 10.5114/jhk/162021

**Published:** 2023-04-20

**Authors:** Michael H. Haischer, Joseph P. Carzoli, Daniel M. Cooke, Joshua C. Pelland, Jacob F. Remmert, Michael. C. Zourdos

**Affiliations:** 1 Department of Exercise Science and Health Promotion, Muscle Physiology Laboratory, Florida Atlantic University, Boca Raton, FL, USA.

**Keywords:** resistance training, muscular endurance, strength, autoregulation

## Abstract

The purpose of this investigation was to determine if average concentric velocity (ACV) of a single repetition at 70% of one-repetition maximum (1RM), ACV of the first repetition of a set to failure at 70% of 1RM, or the velocity loss during the set could predict the number of repetitions performed in the back squat. Fifty-six resistance-trained individuals participated in the study (male = 41, age = 23 ± 3 yrs, 1RM = 162.0 ± 40.0 kg; female = 15, age = 21 ± 2 yrs, 1RM = 81.5 ± 12.5 kg). After 1RM testing, participants performed single repetition sets with 70% of 1RM and a set to failure with 70% of 1RM. ACV was recorded on all repetitions. Regression model comparisons were performed, and Akaike Information Criteria (AIC) and Standard Error of the Estimate (SEE) were calculated to determine the best model. Neither single repetition ACV at 70% of 1RM (R^2^ = 0.004, p = 0.637) nor velocity loss (R^2^ = 0.011, p = 0.445) were predictive of total repetitions performed in the set to failure. The simple quadratic model using the first repetition of the set to failure ($Y=\beta_{0}+\beta_{1}X_{ACVFirst}+\beta_{2}Z+\varepsilon$) was identified as the best and most parsimonious model (R^2^ = 0.259, F = 9.247, p < 0.001) due to the lowest AIC value (311.086). A SEE of 2.21 repetitions was identified with this model. This average error of ~2 repetitions warrants only cautious utilization of this method to predict total repetitions an individual can perform in a set, with additional autoregulatory or individualization strategies being necessary to finalize the training prescription.

## Introduction

Resistance training loads are often prescribed as a percentage of one-repetition maximum (1RM) or with RM zones using repetitions-allowed tables (Haff and Triplett, 2015). Despite the universal application, there is considerable inter-individual variation in the number of repetitions performed at a specific relative intensity (% of 1RM) due to factors such as body mass, femur length, and body fat content (Cooke et al., 2019), along with muscle fiber type composition and training history (Hoeger et al., 1990; Richens and Cleather, 2014; Shimano et al., 2006). If a percentage prescription is used, for example, 4 sets of 8 repetitions at 70% of 1RM, some individuals may fail to complete the prescribed number of repetitions, while others may not receive a sufficient training stimulus. This is consequential as training to failure elongates the recovery period by at least 24 hours compared to non-failure training (Moran-Navarro et al., 2017; Pareja-Blanco et al., 2018) and impairs subsequent performance compared to non-failure training (Vieira et al., 2021), which could in turn harm weekly training frequency and volume. The repetitions in reserve (RIR)-based rating of perceived exertion (RPE) scale (Zourdos et al., 2016), which aims to control for proximity to failure between individuals, could remedy an inappropriate universal load prescription. For example, a training prescription of 4 (sets) × 8 (repetitions) to a 6–8 RPE (i.e., 2–4 RIR), stipulates that an individual chooses a load in which they will have between 2–4 RIR at the end of the set. However, a limitation of the RIR-based RPE scale is that ratings are subjective, which is evidenced by recent data from Zourdos et al. (2021), showing that RPE ratings are not perfectly accurate even for well-trained individuals. Additionally, an athlete could select a load that is too heavy and could inadvertently overshoot the prescribed RPE or even train to failure (Helms et al., 2016; Zourdos et al., 2016), which could result in more fatigue than desired.

Alternatively, velocity-based training (VBT) could be used for the load prescription. An example prescription could be 4 sets of 8 repetitions on the back squat with the final repetition being ≥0.35 m^.^s^-1^ as this is, on average, ending a set at a 2RIR (Zourdos et al., 2016). Additionally, a velocity loss prescription could be used, such as 4 sets, with each being terminated when a specific velocity loss threshold (e.g., 10, 20, 30, or 40% of the first repetition) has been reached (Galiano et al., 2020; Pareja-Blanco et al., 2017; Rodiles-Guerrero et al., 2022; Weakley et al., 2020). However, VBT programming has limitations of its own. Namely, although ending a set around 0.35 m^.^s^-1^ may be 2 RIR for some, this velocity will be even closer to failure for some individuals and further from failure for others. This concept is evidenced by a 95% confidence interval of 0.31–0.44 m·s^-1^ at 0 RIR in trained men in the back squat (Hackett et al., 2021). Similarly, if the first repetition in a set is 0.60 m^.^s^-1^, a 40% velocity loss would terminate the set at the first repetition which reached ≤0.36 m^.^s^-1^, which would result in the same between-individual proximity to failure issue as using absolute velocities. Therefore, the load prescription with VBT is not inherently individualized; instead, individual profiles can be created (Pelland et al., 2022).

A potential additional benefit of VBT is to use the ACV of a warm-up repetition or the ACV of the first repetition of a set to predict performance. Indeed, in a study of tactical athletes, Beckham et al. (2018) examined if a single repetition set or the first repetition in a set to failure could predict total repetitions performed. Those authors observed that repetitions performed to failure during bodyweight pull-ups could be predicted with reasonable accuracy (average prediction error = 2.07 repetitions; R^2^ = 0.841) from the average concentric velocity (ACV) of a single repetition set or the first repetition of a set to failure (Beckham et al., 2018). Similarly, García-Ramos et al. (2018) investigated whether the ACV of the fastest repetition from sets with 60%, 70%, 80%, and 90% of 1RM could accurately predict the number of repetitions to failure in the Smith machine bench press (García-Ramos et al., 2018). Indeed, those authors reported a significant linear relationship (R^2^ = 0.774), but the average prediction error of 3.57 repetitions, nominally larger than that observed by Beckham et al. (2018), suggests that predicting repetitions to failure from ACV may be exercise-specific.

In addition to exercise-specific considerations, the relative intensity used in the studies by Beckham et al. (2018) and García-Ramos et al. (2018) were variable. Therefore, it remains unclear if a single repetition set or the first repetition of a set to failure at a given relative intensity can acutely predict repetitions to failure between subjects. If this is the case, researchers and practitioners could determine daily training loads with a standardized first repetition velocity. Then, a predetermined number of repetitions per set could be prescribed to all participants. This approach would control RIR without the need for individualized velocity profiles, which require sets taken to failure and potentially additional laboratory visits (Pelland et al., 2022). Additionally, this would rectify some of the chief limitations of velocity loss prescriptions often used in the literature; specifically, velocity loss failing to control volume and RIR between and even within participants (Jukic et al., 2022). Furthermore, predicting the number of repetitions performed would be potentially useful in a group setting on an exercise such as the squat where a large interindividual variation of repetitions performed (6–28) has been reported at a given relative intensity (70% of 1RM) (Cooke et al., 2019).

Therefore, the purpose of this study was to determine if the ACV of a single repetition at 70% of 1RM, the ACV of the first repetition of a set to failure at 70% of 1RM, or the percentage velocity loss over the set could predict the number of repetitions performed to failure in the back squat. We hypothesized that the ACV of the fastest single repetition, the first repetition in the set to failure, and the velocity loss during the set would accurately predict the number of repetitions performed in a positive and linear fashion.

## Methods

### Participants

Fifty-eight resistance-trained individuals were recruited for participation (male = 43, female = 15), and further participant details can be seen elsewhere (Haischer et al., 2019). Participants must have performed the back squat an average of 1x/wk for ≥2 yrs and meet minimum strength requirements for inclusion (males: 1RM ≥1.5 x body mass and females: 1RM ≥body mass). Additionally, individuals with contraindications to exercise (e.g., heart disease, serious musculoskeletal disorders, etc.) were excluded and all participants were required to refrain from exercise for 48 hours prior to testing. Participants abstained from stimulants (e.g., caffeine) on the day of testing, but were allowed to eat and drink *ad libitum* throughout the protocol. The Florida Atlantic University Institutional Review Board approved this investigation.

### Measures

#### Body Height, Body Mass, and Body Fat Content

Body height (cm) was measured using a wall-mounted stadiometer (SECA, Hamburg, Germany) and body mass (kg) was assessed via a calibrated digital scale (Mettler-Toledo, Columbus, Ohio, USA). The average sum of two measurements of skinfold thickness acquired from three sites (males: abdomen, front thigh, and chest; females: triceps, suprailiac, and thigh) on the right side of the body was used to estimate body fat content (Jackson and Pollock, 1978). If any site was >2 mm different between measurements, then a third measurement was taken and averaged with the closer of the first two measurements.

#### One-Repetition Maximum (1RM) Testing

Testing for the back squat 1RM was performed in agreement with previously validated procedures (Zourdos et al., 2016). To record the most accurate 1RM, the investigators used the ACV (m^.^s^-1^) recorded via an Open Barbell System Version 2 (OBS2) (Squats & Science, Brooklyn, NY, USA) and each participant reported the RPE according to the RIR-based RPE scale to determine loads for subsequent attempts. Each participant was given five to seven minutes of rest between 1RM attempts. A 1RM was accepted as valid if one of three conditions were met: 1) the participant reported ‘10’ on the RPE scale and the investigator determined an additional attempt with an increased load would be unsuccessful, 2) the participant reported a ‘9.5’ RPE and then proceeded to fail the subsequent attempt with a load increase of 2.5 kg or less, 3) the participant reported an RPE of ≤9 and failed the subsequent attempt with a load increase of 5 kg or less. The squat was performed under the rules and regulations of United States of America Powerlifting, which included a depth of the hip crease passing below the top of the knee (USA Powerlifting, 2021).

#### Velocity Profiling

Participants completed two single repetition squat sets (14 single repetition sets total) at 30%, 40%, 50%, 60%, 70%, 80%, and 90% of 1RM, which were performed as part of a larger study, to establish velocity profiles. Participants were encouraged to lift with maximal intent and at least 2 minutes of rest was given in between each trial. For the purposes of this investigation, only the faster of the two single repetition sets at 70% of 1RM was used for analysis as a predictor of repetitions performed.

#### Repetitions to Failure at 70% of 1RM

Participants were not explicitly instructed to lift with a particular repetition tempo; thus, the concentric intent can be described as habitual. The OBS2 assessed and recorded ACV on all repetitions to observe velocity loss. Volitional failure was determined as the participant either failing a repetition or recording an RPE value of ‘10’ after a successful repetition (Zourdos et al., 2021). The total number of successful repetitions was recorded as the dependent variable. The first repetition ACV and velocity loss were used as predictor variables.

### Design and Procedures

**Figure 1:**
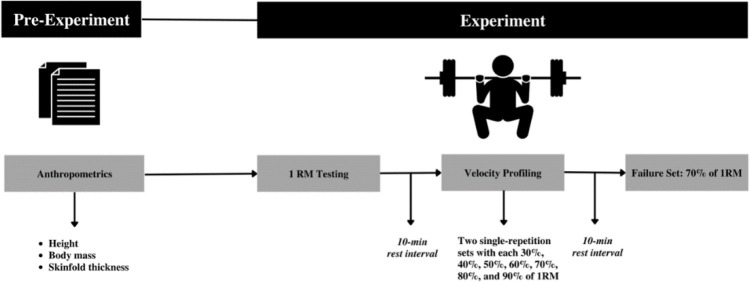
Study Design. 1RM = One-repetition maximum

The aim of this study was to determine if velocity loss or individual repetition ACV could predict repetitions performed in the back squat among trained lifters. Participants reported to the laboratory for one day which began with written informed consent and completion of Health History and Physical Activity Questionnaires. All testing was conducted between the hours of 8 AM and 8 PM at a time that was convenient to participants and in concordance with their usual training habits. This was done to improve the ecological validity of the study because previous data have indicated that strength levels are diminished when individuals train at a time that they are not accustomed to (Sedliak et al., 2008). Upon completion of the questionnaires, participants underwent anthropometric assessments before performing a five-minute dynamic warm-up and squat 1RM testing. Squat 1RM testing was performed in accordance with previously validated procedures (Zourdos et al., 2016). Next, participants had a 10-min rest period prior to completing velocity profiling sets. Finally, participants then rested additional 10 minutes before completing one set to volitional failure with 70% of 1RM on the back squat. The completed study procedures can be seen in Figure 1.

**Figure 2:**
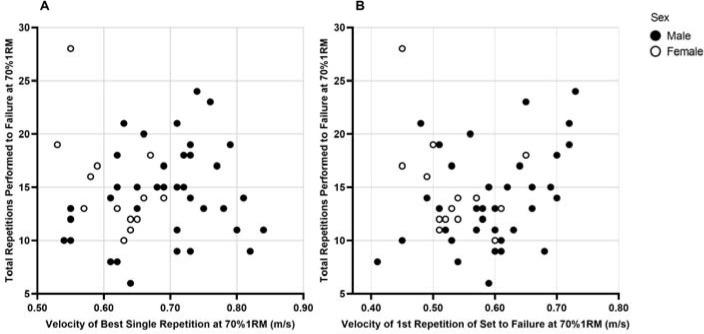
Relationship Between Total Repetitions Performed at 70% of 1RM and the Average Concentric Velocity. The associations between repetitions performed and the velocity of a separate, single repetition at 70%1RM (A), and the velocity of the first repetition of the set to failure (B) were not significant (p > 0.05)

### Statistical Analysis

The independent variables used for analysis in this study were sex, the ACV of the best (faster) single repetition effort at 70% of 1RM during velocity profiling (X_ACVBest_), the ACV of the first repetition during the set to failure (X_ACVFirst_), and the velocity loss from the first-to-last repetitions of the set to failure expressed as a percentage (X_VeloLoss_). First, descriptions of all continuous variables of interest were produced. To assess normality, Kolmogorov-Smirnov and Shapiro-Wilk tests were conducted. Additionally, the Breusch-Pagan test was performed for all three X-variables to investigate violations of the homogeneity of the variances assumption. Total repetitions performed were discovered to be abnormally distributed, thus bootstrapped Spearman’s rho correlations with 1000 replicate samples were produced between ACV independent variables (X_ACVBest_, X_ACVFirst_, and X_VeloLoss_) and total repetitions. A variety of model comparisons were then used to elucidate the best and most parsimonious predictive model of the number of successful repetitions that a participant could perform.

Initially, linear regression models were run for each of the ACV variables with total repetitions serving as the outcome variable $\left(Y=\beta_{0}+\beta_{1}X+\varepsilon \right)$. Models for X_ACVBest_ and X_VeloLoss_ were performed with ordinary least squares estimation, while the model for X_ACVFirst_ was done using weighted least squares estimation as the homogeneity of variances assumption was violated. Of those three models, the most promising variable (X_ACVFirst_) was identified using R^2^ values and used for additional analysis. Next, additional exploratory models were created to adjust for sex (Z variable; $Y=\beta_{0}+\beta_{1}X_{ACVFirst}+\beta_{2}Z+\varepsilon$) and the sex-ACV variable interaction effect ($Y=\beta_{0}+\beta_{1}X_{ACVFirst}+\beta_{2}Z+\beta_{3}X_{ACVFirst}Z+\varepsilon$). As the appearance of the scatter plot suggested a linear model might not be sufficient to represent the data, a quadratic trend analysis was also conducted on the simple ($Y=\beta_{0}+\beta_{1}X_{ACVFirst}+\beta_{2}X_{ACVFirst}^{2}+\varepsilon$), adjusted ($Y=\beta_{0}+\beta_{1}X_{ACVFirst}+\beta_{2}X_{ACVFirst}^{2}+\beta_{3}Z+\varepsilon$), and multiplicative models ($Y=\beta_{0}+\beta_{1}X_{ACVFirst}+\beta_{2}X_{ACVFirst}^{2}+\beta_{3}Z+\beta_{4}X_{ACVFirst}Z+\varepsilon$). In the end, to allow for selection of the most parsimonious model of repetitions performed, Akaike Information Criteria (AIC) were hand-calculated for each model using the formula 𝐴𝐼𝐶=−2𝐿𝑜𝑔𝐿𝑖𝑘𝑒𝑙𝑖ℎ𝑜𝑜𝑑 × 2𝑝, with *p* as the number of predictor variables (Akaike, 1974). With the exception of AIC, all analyses were performed with SPSS version 25.0 (IBM Corp., Armonk, NY, USA) and the level of significance was set at *p* ≤ 0.05.

## Results

### Participants’ Characteristics and Evaluation of Model Assumptions

Of the 58 participants that volunteered for the study, 56 completed the back squat set to volitional failure at 70% of 1RM in which velocity analysis could be successfully conducted. Descriptive statistics of the participants and mean data for repetitions performed, first and best repetition ACV, and velocity loss are presented in Table 1. Scatter plots of total repetitions performed and individual repetition velocity variables, X_ACVBest_ and X_ACVFirst_, are presented in Figure 1.

**Table 1:** Descriptive Statistics.

N = 56	Age (yrs)	1RM (kg)	BMI	Height (cm)	Body Mass (kg)	Body Fat (%)
Mean ± SD	23 ± 3	140 ± 50	26.67 ± 3.75	172.6 ± 8.8	80.25 ± 16.6	10.94 ± 3.44
				**Mean ± SD**	**Minimum**	**Maximum**
Total Repetitions Performed at 70% of 1RM				14 ± 4	6	28
ACV of the Best Single Repetition at 70% of 1RM (X_ACVBest_)				0.667 ± 0.078	0.53	0.84
ACV of the First Repetition of the Set to Failure at 70% of 1RM (X_ACVFirst_)				0.579 ± 0.076	0.41	0.73
Percent Velocity Loss During the Set to Failure at 70% of 1RM (X_VeloLoss_)				46.5 ± 13.3	12	73

1RM = One Repetition Maximum; ACV = Average Concentric Velocity; BMI = Body Mass Index

Results of the tests of normality and for heteroscedasticity are shown in Table 2. The total number of repetitions performed was not normally distributed. Additionally, the Breusch-Pagan test of the velocity of the first repetition in the set to failure (X_ACVFirst_) indicated a violation of the homogeneity of variances assumption, dictating weighted least squares estimation of regression models.

**Table 2:** Tests of Normality and Heteroscedasticity.

	Kolmogorov-Smirnov			Shapiro-Wilk			Breusch-Pagan		
	Statistic	df	Sig.	Statistic	df	Sig.	Chi-Square	df	Sig.
Total Repetitions Performed at 70% of 1RM	0.13	56	0.02	0.959	56	0.056			
ACV of the Best Single Repetition at 70% of 1RM (X_ACVBest_)	0.104	56	0.2	0.971	56	0.198	0.038	1	0.846
ACV of First Repetition of the Set to Failure at 70% of 1RM (X_ACVFirst_)	0.089	56	0.2	0.983	56	0.623	4.116	1	0.042
Percent Velocity Loss During the Set to Failure at 70% of 1RM (X_VeloLoss_)	0.08	56	0.2	0.982	56	0.576	0.227	1	0.599

1RM = One Repetition Maximum; ACV = Average Concentric Velocity

Due to the violation of normality, Spearman’s rho correlations were used to evaluate the association between repetitions performed and independent variables. Bootstrapping with 1000 replicate samples was also implemented to increase the robustness of the analyses and allow for reporting of bias corrected and accelerated (BCa) 95% confidence intervals. The values of these correlations are shown in Table 3.

**Table 3:** Correlations Between Total Repetitions Performed and Velocity Variables.

	Correlation Rho	Sig.	Bias	Std. Error	BCa 95% Confidence Interval	
					Lower	Upper
ACV of the Best Single Repetition at 70% of 1RM (X_ACVBest_)	0.145	0.286	0.001	0.142	−0.123	0.403
ACV of the First Repetition of the Set to Failure at 70% of 1RM (X_ACVFirst_)	0.173	0.203	0.003	0.155	−0.141	0.476
Percent Velocity Loss During the Set to Failure at 70% of 1RM (X_VeloLoss_)	0.148	0.276	−0.003	0.143	−0.14	0.406

* Based on 1000 Bootstrap Samples; 1RM = One Repetition Maximum; ACV = Average Concentric Velocity; BCa = Bias Corrected and Accelerated

### Regression Model Comparisons

Despite the absence of significant correlations (Table 3), variables were analyzed in regression models to more accurately assess their predictive capabilities. As can be seen in Table 4, an initial linear model comparison to identify the most promising X-variable was performed, with models specified as $Y=\beta_{0}+\beta_{1}X+\varepsilon$. While the single repetition during velocity profiling (X_ACVBest_) and velocity loss (X_VeloLoss_) were not significant predictors of repetitions performed, the first repetition of the set to failure (X_ACVFirst_) was identified as a significant predictor and was used as the primary independent variable in all models moving forward.

**Table 4:** Linear Model Comparison Between Velocity Independent Variables.

Model	R^2^	S.E.E.	SSreg	SSres	F	Sig.
$Y=\beta_{0}+\beta_{1}X_{ACVFirst}+\varepsilon$	0.004	4.386	4.333	1038.649	0.225	0.637
$Y=\beta_{0}+\beta_{1}X_{ACVFirst}+\varepsilon$	0.086	2.434	30.2	319.893	5.098	0.028
$Y=\beta_{0}+\beta_{1}X_{ACVFirst}+\varepsilon$	0.011	4.371	11.303	1031.679	0.592	0.445

S.E.E. = Standard Error of the Estimate

Additional model comparisons were then performed between the simple ( $Y=\beta_{0}+\beta_{1}X_{ACVFirst}+\varepsilon$), adjusted (for sex; Z variable; $Y=\beta_{0}+\beta_{1}X_{ACVFirst}+\beta_{2}Z+\varepsilon$), and multiplicative (X_ACVFirst_ and sex interaction; $Y=\beta_{0}+\beta_{1}X_{ACVFirst}+\beta_{2}Z+\beta_{3}X_{ACVFirst}Z+\varepsilon$) models, with quadratic trends also analyzed. Quadratic trend analysis with the additional component (X^2^_ACVFirst_) was performed as the scatter plots (Figure 1) suggested that a linear trend might not sufficiently represent the data. The complete model comparison can be seen in Table 5, with parameter estimates of these models shown in Table 6. Overall, the simple quadratic model ($Y=\beta_{0}+\beta_{1}X_{ACVFirst}+\beta_{2}X_{ACVFirst}^{2}+\varepsilon$) was identified as the best and most parsimonious model due to the lowest AIC value (311.086).

**Table 5:** Weighted Least Squares Comparison Between X_ACVFirst_ Models.

Model	R^2^	S.E.E.	F	Sig.	AIC
Simple	0.086	2.434	5.098	0.028	320.75
Adjusted	0.095	2.444	2.795	0.07	322.332
Multiplicative	0.149	3.123	3.032	0.037	319.556
Simple Quadratic	0.259	2.213	9.247	<0.001	311.086
Adjusted Quadratic	0.267	2.222	6.304	0.001	312.544
Multiplicative Quadratic	0.278	2.226	4.915	0.002	313.744

AIC = Akaike Information Criterion

**Table 6:** Parameterization of Repetitions Performed and X_ACVFirst_ Models.

Model	Parameter	Estimate	Std. Error	Sig.
Simple	β_0_	4.33	4.5737	0.344
	β_1_	17.002	7.53	0.028
Adjusted	β_0_	3.747	4.625	0.421
	β_1_	19.355	8.217	0.022
	β_2_	−1.065	1.454	0.467
Multiplicative	β_0_	28.089	10.902	0.013
	β_1_	−25.361	20.131	0.213
	β_2_	−28.074	12.109	0.024
	β_3_	49.064	21.911	0.029
Simple Quadratic	β_0_	102.408	28.238	0.001
	β_1_	−317.943	95.645	0.002
	β_2_	281.533	80.187	0.001
Adjusted Quadratic	β_0_	101.545	28.377	0.001
	β_1_	−314.658	96.137	0.002
	β_2_	280.662	80.525	0.001
	β_3_	−0.996	1.322	0.455
Multiplicative Quadratic	β_0_	100.585	28.447	0.001
	β_1_	−296.503	98.384	0.004
	β_2_	250.821	87.159	0.006
	β_3_	−12.317	12.608	0.333
	β_4_	20.392	22.585	0.371

## Discussion

The initial findings of this study were contrary to the hypothesis, in that bootstrapped Spearman’s rho correlations did not find a significant linear relationship between ACV of the faster single repetition, the first repetition in the set, or velocity loss with repetitions performed. However, the regression models did show that first repetition ACV significantly predicted repetitions performed, while single repetition ACV and velocity loss did not (Table 4). To that end, X_ACVFirst_ was used in the adjusted and multiplicative models (Tables 5 and 6). Since the Breusch-Pagan test indicated that the relation between X_ACVFirst_ and total repetitions was heteroscedastic, weighted least squares estimation was used for regression analyses using this independent variable. The adjusted model accounted for the addition of sex, and the multiplicative model included sex and the interaction between sex and ACV_First_. Furthermore, the Akaike Information Criterion and standard error of the estimate suggested that the simple model with a quadratic trend was the most plausible (Table 5). This model showed a prediction error of 2.21 repetitions (SEE = 2.21), which is similar to the prediction error of 2.07 reported by Beckham et al. (2018). Thus, the results of this study suggest that exploratory non-linear prediction models using first repetition velocity may be able to accurately predict the number of repetitions to failure at the same relative intensity across individuals.

The prediction error of ~2 repetitions in the present study is nominally lower than the prediction error of ~3.6 repetitions reported in both the Smith Machine bench press by Garcia-Ramos et al. (2018) and the Smith Machine prone bench pull by Miras-Moreno et al. (2022). Discrepancies in the study design may contribute to this difference in prediction error, but the most likely contributions are differences in the prediction model used. Specifically, the mentioned studies used simple linear models with the predictor variable as the fastest repetition velocity of the set (often the first repetition). In the present study, linear models were also conducted, but the first repetition velocity was considerably less associated with repetition performance in comparison to these studies (R^2^ = 0.086 vs. R^2^ = 0.700−0.774). Thus, it may be that the prediction errors reported by Garcia-Ramos et al. (2018) and Miras-Moreno et al. (2022) could be improved by utilizing non-linear regression models as in the present study.

Interestingly, Beckham et al. (2018) reported ACV_First_ to predict repetitions performed to a similar degree of accuracy as the present study, despite using a linear regression like Garcia-Ramos et al. (2018) and Miras-Moreno et al. (2022). We postulate two reasons for the similar degree of prediction accuracy in Beckham et al. (2018) despite different prediction models. First, Beckham et al. (2018) instructed subjects to use maximal concentric intent on the first repetition, which has been reported to improve the ability of velocity to predict 1RM (Macarilla et al., 2022). Secondly, Beckham et al. (2018) had subjects begin each pullup repetition from a “dead hang” position; thus, each repetition began with the concentric phase rather than the eccentric phase as in a back squat. In an exercise, such as the pullup with minimal skill requirement, which begins with the concentric phase, it is likely that velocity will decline linearly each repetition, especially when using maximal intent on each repetition. For these reasons, a linear regression was likely an appropriate prediction model in Beckham et al. (2018). However, in the present study, the use of an exercise with greater technical skill that began with the eccentric phase coupled with the lack of a maximal intent cue, likely led to the lack of a linear decline in velocity. Therefore, the appropriate method to predict total repetitions from ACV_First_ may be both exercise- and intent-specific.

Due to the lack of a maximal concentric intent cue in the present study, it seems that different pacing strategies were used (Halperin et al., 2014), as evidenced by a wide range of velocity loss (12–73%) during the set to failure at 70% of 1RM. Individuals that experienced minimal velocity loss during the set may have paced themselves from the beginning, trying to conserve their energy to complete as many repetitions as possible. This hypothesis of less than maximal effort during the first repetition of the set to failure is supported by the difference in ACV between the best single repetition at 70% of 1RM (0.667 m^.^s^-1^) and the ACV of the first repetition on the failure set, also at 70% of 1RM (0.579 m^.^s^-1^). On the other hand, participants with greater velocity loss may have focused on moving the barbell as fast as possible on each repetition. Even so, it appears that the number of total repetitions performed was not impacted by the strategy used, as velocity loss was not a significant predictor of repetition performance. While the lack of a maximal concentric intent cue may enhance the ecological validity of our analysis, it may have harmed predictive ability and explain the discrepant findings. It is also possible that first repetition ACV was slower on the failure set then the single repetition sets at 70% of 1RM due to potential fatigue accumulation from the 14 single repetition sets performed between 30–90% of 1RM prior to the failure set. However, as previously reported (Cooke et al., 2019), subjects performed 14 ± 4 repetitions during the 70% to failure set in this study, which is considerably higher than the typically reported (Haff et al., 2016) number of repetitions to failure. Ultimately, it cannot be fully ascertained whether accumulated fatigue or a lack of a maximal intent cue is responsible for the slower first repetition velocity during the failure set, and the potential impact of the 14 single repetition sets on the first repetition velocity of the failure set is a limitation of this study. However, since the number of repetitions to failure are higher than previously reported, it seems that the lack of a maximal intent cue was at least partially responsible.

Finally, the relative intensity used in the study by Beckham et al. (2018), in comparison to our study, is undoubtedly more variable. To explain, the back squat exercise in the current study was standardized to 70% of a tested 1RM, while subjects in Beckham et al. (2018) performed bodyweight pull-ups, which likely resulted in a wide range of relative pull-up intensities. In other words, although each subject was lifting their own bodyweight, the load relative to their 1RM lifted was not standardized across the sample. This range of relative intensities in and of itself likely contributed to the prediction accuracy, ultimately leading to similar prediction accuracy as the present study despite the use of a linear model.

Future work in this area should draw on the strengths of the current study and previous studies using a maximal concentric intent cue, a consistent relative intensity, and various non-linear prediction models. If the velocity of a single repetition is reliably related to total repetitions performed at a given relative intensity across individuals, it could be used as a load prescription tool to control RIR and ensure an appropriate training stimulus for each session. This would avoid the necessity of individual-level velocity profiles and be of high practical utility, especially in group settings such as teams. However, whether a robust relationship exists across individuals requires further investigation.

## Conclusions

The current study indicates that ACV_First_ during a set to failure at 70% of 1RM in the back squat can predict the total number of repetitions performed to failure with reasonable accuracy (within ~2 repetitions). Thus, non-linear prediction models have the potential to provide high prediction accuracies of repetition performance from first repetition velocity. However, from a practical application perspective, additional research should first explore these models and whether they are reliable with different concentric cueing (i.e., maximal intent and habitual intent), in exercises beyond the back squat, at different relative intensities, and across time (e.g., beginning and end of a training program). Additionally, we suggest that future studies utilize multiple regression models using other variables which may affect the velocity-repetition relationship to predict acute repetitions performed.
